# MAKING AGE-INCLUSIVE CAMPUS PRACTICES MORE DEMENTIA-INCLUSIVE

**DOI:** 10.1093/geroni/igae098.1934

**Published:** 2024-12-31

**Authors:** Joann Montepare, Katherina Nikzad-Terhune

**Affiliations:** Chair; Discussant

## Abstract

Shifting demographics are calling for higher education to respond to aging populations through more age-inclusive programs, practices, and partnerships. As institutions navigate this new educational terrain, gaps are evident. This symposium draws attention to the need to integrate age-inclusive with dementia-inclusive efforts. Addressing this need with educational interventions can benefit our students’ own healthy aging and longevity. Moreover, efforts that involve older adults living with dementia likewise have beneficial capacity for their well-being. To begin, Montepare (Lasell University) will provide an overview of contemporary age-inclusive initiatives in higher education (Age-Friendly University/AFU, Age Inclusivity Domains of Higher Education/AIDHE) and research motivating dementia-focused educational opportunities. Next, Mastel-Smith and colleagues (University of Texas at Tyler) will describe how nursing students co-created life stories online with people living with dementia who served as mentors and will show how qualitative data from students and mentors revealed positive outcomes for both groups. Kimzey (Texas Christian University) will then discuss a novel educational strategy in which a person with dementia (expert by experience) participated in teaching an undergraduate dementia course for health profession students and present data from focus groups and interviews showing the benefits of dementia-inclusive educational design. Finally, Shovali (Eckerd College) will describe how harnessing technology can expand dementia-inclusive teaching and learning. Specifically, how incorporating oculus VR headsets in experiential classroom activities can enhance students’ understanding and empathy of people living with dementia. Katherina Nikzad-Terhune (Northern Kentucky University), co-convener of the Age Inclusivity in Higher Education Interest Group, will serve as discussant.

